# Proteomic Resistance Biomarkers for PI3K Inhibitor in Triple Negative Breast Cancer Patient-Derived Xenograft Models

**DOI:** 10.3390/cancers12123857

**Published:** 2020-12-21

**Authors:** Zhanfang Guo, Tina Primeau, Jingqin Luo, Cynthia Zhang, Hua Sun, Jeremy Hoog, Feng Gao, Shixia Huang, Dean P. Edwards, Sherri R. Davies, Rebecca Aft, Li Ding, Matthew J. Ellis, Shunqiang Li, Cynthia X. Ma

**Affiliations:** 1 Division of Oncology, Department of Medicine, Washington University School of Medicine, St. Louis, MO 63110, USA; zguo@wustl.edu (Z.G.); tprimeau@wustl.edu (T.P.); cynthiazhang@wustl.edu (C.Z.); jhoog@wustl.edu (J.H.); sdavies@wustl.edu (S.R.D.); 2 Division of Public Health Science, Siteman Cancer Center Biostatistics Core, Washington University School of Medicine, St. Louis, MO 63110, USA; jingqinluo@wustl.edu (J.L.); feng@wustl.edu (F.G.); 3 Department of Medicine, McDonnell Genome Institute, Siteman Cancer Center, Washington University School of Medicine, St. Louis, MO 63108, USA; hua.sun@wustl.edu (H.S.); lding@wustl.edu (L.D.); 4 Dan L. Duncan Cancer Center and Department of Molecular and Cellular Biology, Baylor College of Medicine, Houston, TX 77030, USA; shixiah@bcm.edu (S.H.); deane@bcm.edu (D.P.E.); 5 Department of Surgery, Washington University School of Medicine, St. Louis, MO 63110, USA; aftr@wustl.edu; 6 Lester and Sue Smith Breast Center, Dan L. Duncan Comprehensive Cancer Center and Departments of Medicine and Molecular and Cellular Biology, Baylor College of Medicine, Houston, TX 77030, USA; matthew.ellis@bcm.edu

**Keywords:** biomarkers, PI3K inhibitor, BKM120, triple negative breast cancer, patient-derived xenograft

## Abstract

**Simple Summary:**

The objective of this study is to identify potential proteomic biomarkers in triple negative breast cancer (TNBC) that associate with response to PI3K inhibitors which are in clinical trials. We tested a panel of TNBC patient-derived xenograft (PDX) models for their tumor growth response to a pan-PI3K inhibitor, BKM120. Proteomic analyses by reverse phase protein array (RPPA) of 182 markers were performed on baseline and post short-term treatment PDX samples, to correlate with tumor growth response. We identified several baseline and treatment induced proteomic biomarkers in association with resistance. These results provide important insights for the development of PI3K inhibitors in TNBC.

**Abstract:**

PI3K pathway activation is frequently observed in triple negative breast cancer (TNBC). However, single agent PI3K inhibitors have shown limited anti-tumor activity. To investigate biomarkers of response and resistance mechanisms, we tested 17 TNBC patient-derived xenograft (PDX) models representing diverse genomic backgrounds and varying degrees of PI3K pathway signaling activities for their tumor growth response to the pan-PI3K inhibitor, BKM120. Baseline and post-treatment PDX tumors were subjected to reverse phase protein array (RPPA) to identify protein markers associated with tumor growth response. While BKM120 consistently reduced PI3K pathway activity, as demonstrated by reduced levels of phosphorylated AKT, percentage tumor growth inhibition (%TGI) ranged from 35% in the least sensitive to 84% in the most sensitive model. Several biomarkers showed significant association with resistance, including elevated baseline levels of growth factor receptors (EGFR, pHER3 Y1197), PI3Kp85 regulatory subunit, anti-apoptotic protein BclXL, EMT (Vimentin, MMP9, IntegrinaV), NFKB pathway (IkappaB, RANKL), and intracellular signaling molecules including Caveolin, CBP, and KLF4, as well as treatment-induced increases in the levels of phosphorylated forms of Aurora kinases. Interestingly, increased AKT phosphorylation or PTEN loss at baseline were not significantly correlated to %TGI. These results provide important insights into biomarker development for PI3K inhibitors in TNBC.

## 1. Introduction

Triple negative breast cancer (TNBC) is a significant clinical challenge due to its aggressive clinical course and frequent resistance to chemotherapy [1]. Developing molecularly targeted therapeutics is an unmet need, however this effort has been challenged by the significant inter-tumor heterogeneity of this disease and the difficulty of obtaining sufficient tumor material in clinical trials to identify predictive biomarkers and resistance mechanisms [2,3]. Patient-derived xenograft (PDX) models capture the mutational profiles and the molecular heterogeneity of human breast cancer, providing a robust preclinical platform for this purpose [4,5,6,7,8,9]. In this study, we evaluated the efficacy and response biomarkers of therapeutic targeting PI3K in a panel of TNBC PDX models.

PI3K signaling plays key regulatory roles in many cellular processes, including cell survival, proliferation, differentiation, and angiogenesis [10,11]. Hyperactivation of the PI3K/AKT pathway has been associated with TNBC [12,13], often as a result of *PTEN* mutation/loss (35%), and less frequently, mutations in *PIK3CA* (7%) [12,14], which theoretically should generate sensitivity to PI3K inhibitors [15]. Additionally, the importance of the PI3K pathway in the tumorigenesis of TNBC is supported by the preclinical observation that PTEN inactivation leads to “basal-like” breast cancer in animal models [16,17]. However, single agent PI3K inhibition has shown limited efficacy in TNBC [18], and the resistance mechanisms are not fully understood. The pan-PI3K inhibitor BKM120 that was chosen for this study has been shown to induce a partial response in a patient with TNBC in the initial phase I study [19] and targets all of the class I PI3-kinase isoforms (p110α/β/δ/γ) [20]. We hypothesized that a subset of TNBC is growth-dependent on PI3K signaling and proteomic analysis of PDX models at baseline, and following short-term treatment could identify candidate biomarkers predictive of growth response to BKM120.

## 2. Results

### 2.1. Generation and Characterization of TNBC PDX Models

To investigate the response and resistance mechanisms to PI3K inhibition, we selected the first 17 sequential TNBC PDX models available at the Washington University PDX core. Ten of the 17 models have been reported previously, including WHIMs 2, 4, 5, 6, 12, 13, 14, 21, 25, 30 [6,7,8]. Table S1 lists the clinical characteristics and treatment history of patients before and after providing the samples for PDX engraftment. Five models were derived from African American women. Sixteen of the 17 PDX models (with the exception of WHIM30) were derived from patients with lethal TNBC that eventually claimed their lives. The median disease-free survival (DFS) was 10.3 months, and the median overall survival (OS) was 37 months from the time of initial diagnosis for patients from whom these PDX models were derived. All PDX models were from biopsies that confirmed TNBC, although the patient who provided the sample for establishing WHIM4 had an initial HER2-positive disease prior to recurrence, and the patient who provided the sample for establishing WHIM31 had an initial ER positive and HER2 negative disease. Seven PDX models were derived from treatment-naïve biopsies from patients with locally advanced (WHIMs 2, 12, 30), at initial metastatic recurrence (WHIM34) or de novo stage IV disease (WHIMs 3, 6, 29), while others from residual disease post neoadjuvant chemotherapy (WHIM21) or a metastatic site during the course of disease (WHIMs 4, 5, 10, 13, 14, 25, 31, 34, 36, 48). WHIM2 (primary breast) and WHIM5 (brain metastasis) were derived from the same patient, a 44-year-old African American woman with TNBC, reported previously [7]. Patients whose tumors were established in WHIM30 and WHIM31 carried germline *BRCA1* mutations.

**Figure 1:**
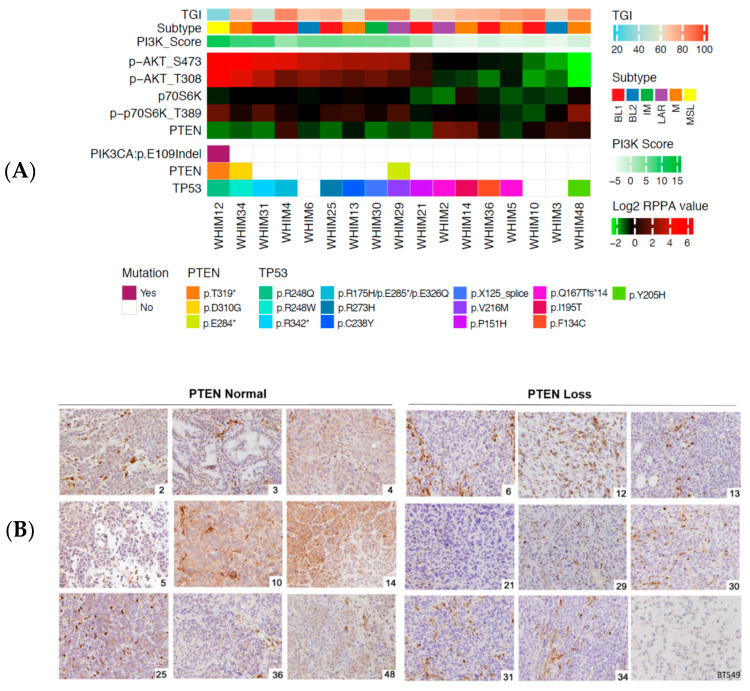
Genomic characterization and proteomic analysis of PI3K pathway. Panel (**A**) shows the heatmap illustrating somatic mutations of TP53 and key PI3K pathway genes, TNBC subtypes (of later passage PDX), and quantitation of selected PI3K pathway downstream signaling components by RPPA. PI3K_Score is the sum of the phosphoprotein levels of Akt, mTOR, GSK3, S6K, and S6, minus the total levels of pathway-inhibitor PTEN, as previously described (9). TGI, % tumor growth inhibition by BKM120 compared to vehicle, is also indicated for each PDX model to contrast with the baseline tumor characteristics. Panel (**B**) shows representative pictures of IHC for PTEN for each PDX model (WHIM number is located at the right lower corner of each picture). PTEN null or loss is observed in WHIM6, 12, 13, 21, 29, 30, 31, 34. PTEN expression is observed in WHIM2, 3, 4, 5, 10, 14, 25, 36, 48. PDX ID corresponding to WHIM numbers are annotated at the right lower corner for each model. Staining for BT549 (PTEN null negative control) is also included. MCF7 was used as positive control for PTEN IHC.

We demonstrated in previous reports that included 10 of the 17 PDX models that they preserve the genomic alterations and gene expression profiles of the original tumor and showed stability in the genomic and proteomic characteristics across the early passages tested [6,7,21]. Additionally, these PDX models represent the diverse inter-tumor heterogeneity of human TNBC in gene expression and proteomic profiles [6,8]. For this study, we performed whole exome sequencing for all 17 PDX models along with the corresponding patient tumor and germline DNA. The somatic mutations were highly conserved between the PDX models and their human tumor counterpart (Figure S1). Table 1 lists selected potential actionable mutations and genomic alterations in the PI3K pathway genes. These PDX models were further characterized by mRNA expression profiling with whole genome microarray, reverse phase protein array (RPPA) analysis of 182 markers, and immunohistochemistry for PTEN. Figure 1 illustrates PI3K pathway genomic and proteomic alterations identified in the PDX models.

TNBCtype analysis of the microarray gene expression data indicated relative stability between human and early passage PDX tumors in (basal-like) BL1 (both remained the same) and mesenchymal (M) subtypes (four remained the same, WHIM5 was not able to be typed in PDX) (Table S2). However, the mesenchymal stem-like (MSL) subtype and the immune (IM) subtype tend to switch to other subtypes during PDX engraftment. For example, among the three human tumors with MSL subtypes, one switched to LAR and two switched to BL1 as early as passage 1 of PDX engraftment. Among the three human tumors with IM subtypes, one switched to BL2 after passage 1, one switched to MSL at passage 1, and one to M at passage 1. BL1 is the most frequent eventual subtype observed in the PDX models (*n* = 6), followed by M (*n* = 3), LAR (*n* = 2) and BL2 (*n* = 2).

PI3K pathway activities among the PDX models were assessed by proteomic analysis with RPPA. To avoid batch effect, RPPA for all baseline untreated PDX models were tested in a single batch of 182 markers (Table S3). In addition, immunohistochemistry (IHC) for PTEN was performed. Eight models were negative for PTEN expression by IHC (WHIM 6, 12, 13, 21, 29, 30, 31, and 34), all of which showed low protein expression by RPPA analysis except WHIM13 (Figure 1A,B). PTEN loss of expression was associated with increased levels of PI3K signaling by RPPA PI3K score and by the levels of phosphorylated AKT and S6K (Figure 1A).

### 2.2. Tumor Growth Response to BKM120 in TNBC PDX Models

**Figure 2:**
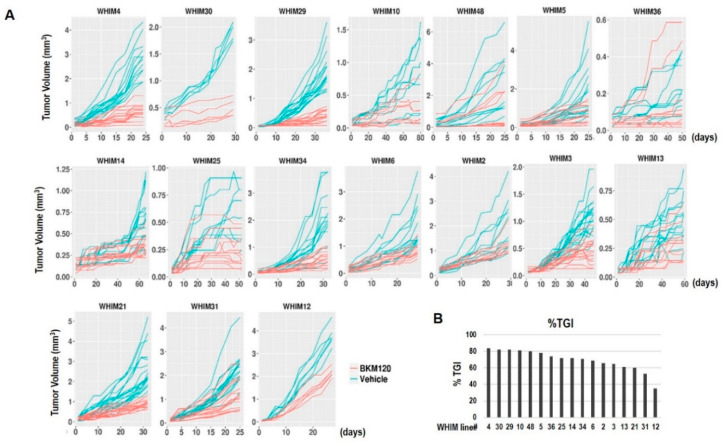
PDX tumor growth response to BKM120. Panel (**A**) shows tumor volume changes of individual PDX models over time in response to treatment with either vehicle or BKM120 30 mg/kg orally days 1–5 each week. Each line represents a single tumor. Panel (**B**) shows the bar graph of the percentage tumor growth inhibition (%TGI) at the completion of the experiments for each PDX model.

We have shown previously that responses to PI3K inhibition with BKM120 in TNBC PDX models are highly variable [22]. In this study, we included additional TNBC PDX models, for a total of 17 PDX models for better assessment of tumor response and evaluation of predictive biomarkers and resistance mechanisms. Figure 2 shows that BKM120 therapy led to significant tumor growth inhibition in all models, with percentage of tumor growth inhibition (%TGI) ranging from 35% in the least sensitive model WHIM12 and 84% in the most sensitive model WHIM4. Although tumor shrinkage was not observed for any of the models, five of the 17 PDX models (23.5%), WHIM 10, 29, 30, 4, and 48 achieved at least 80% TGI in response to BKM120.

### 2.3. RPPA Analysis Indicating PI3K Pathway Inhibition by BKM120

**Figure 3:**
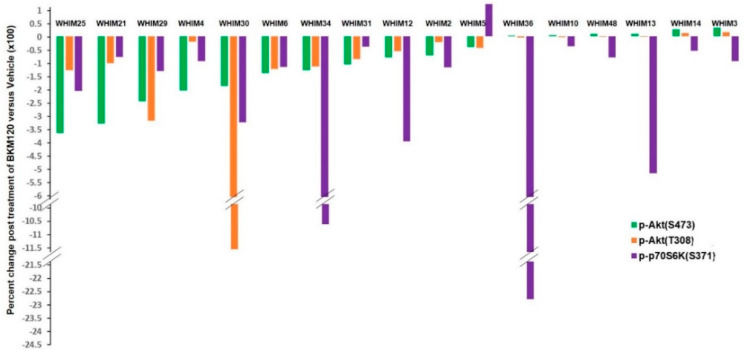
BKM120 induced inhibition of PI3K pathway signaling in TNBC PDX models.

To assess treatment-induced changes in oncogenic signaling pathways, we collected PDX tumors following 3 days of daily administration of BKM120 or vehicle and subjected the tumor lysates to RPPA analysis that included 182 markers (Table S3). Multiple markers were significantly altered in each of the PDX models. Significant inhibition of PI3K pathway signaling, as demonstrated by decreased levels of phosphorylated forms of AKT and S6K, was observed following BKM120 compared to vehicle across different PDX models (Figure 3 and Table S4).

### 2.4. Baseline Proteomic Analysis Reveals Potential Resistance Biomarkers to BKM120

We first investigated whether any baseline biomarkers correlated with tumor growth response to BKM120 as measured by %TGI. Because of the small number of PDX models that carry mutations in PI3K pathway genes, we could not correlate mutations with response. We performed Spearman’s correlation of expression levels of baseline protein markers by RPPA with %TGI and identified several markers that are negatively correlated with %TGI, including growth factor receptors (EGFR, pHER3 Y1197), PI3Kp85 regulatory subunit, anti-apoptotic protein BclXL, EMT (Vimentin, MMP9, IntegrinaV), NFKB pathway (IkappaB, RANKL), and intracellular signaling molecules including Caveolin, CBP, and KLF4 (Table 2, Figure S2). As a proof of concept that targeting the identified resistance biomarkers could improve the cytotoxicity of BKM120, we assessed the in vitro cytotoxic response of two TNBC cell lines, WHIM3 and MDA-MB 231, to an EGFR inhibitor and BKM120, either alone or in combination. The combination index was below 1, indicating synergism between BKM120 and the EGFR inhibitor tested (erlotinib or neratinib in WHIM3; neratinib in MDA-MB 231) in both cell lines (Figure S3). Levels of proapoptotic markers (Bak and Caspase 3) were positively correlated with %TGI (Table 2, Figure S2). Interestingly, markers indicating PI3K pathway signaling activation, including levels of PTEN and phosphorylated PI3K downstream components, were not significantly correlated to %TGI (Table S5).

The percent change in RPPA quantitation of indicated markers, including pAKT(S473), pAKT(T308), and pS6K, is shown for each PDX model following 3 days of either BKM120 (*n* = 3) or vehicle treatment (*n* = 3). Percent change = (mean value post BKM120—mean value post vehicle)/mean value post vehicle.

### 2.5. Treatment-Induced Proteomic Changes Reveal Potential Resistance Biomarkers to BKM120

**Figure 4:**
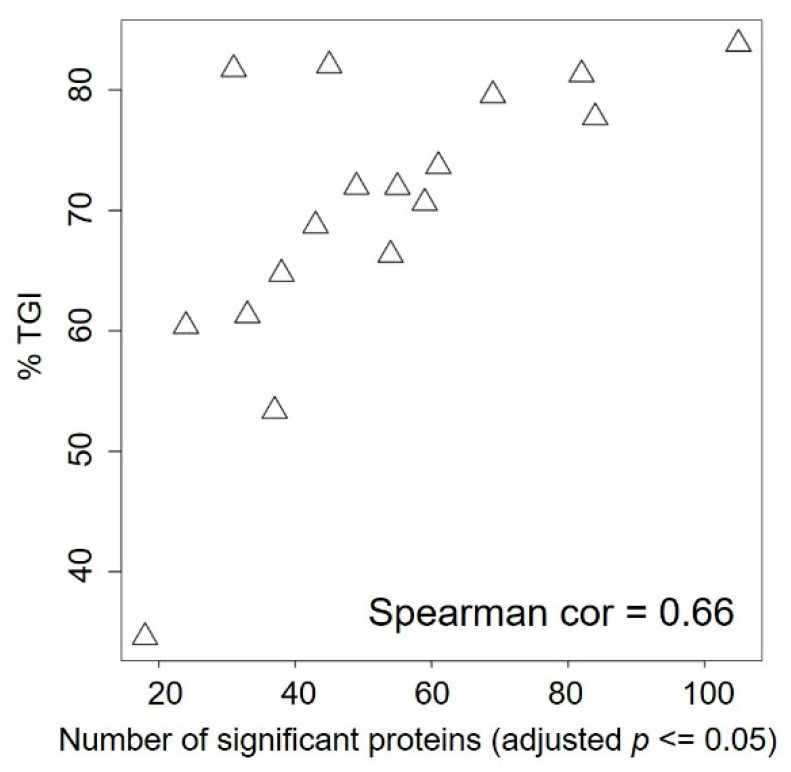
Scatter plot correlating %TGI with the number of significantly changed proteins comparing BKM120 vs. vehicle therapy.

**Figure 5:**
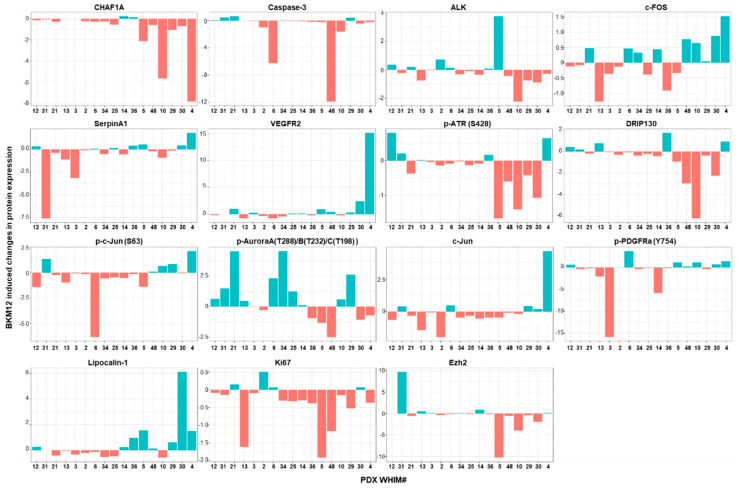
Barplot of RPPA proteins with expression change significantly correlated TGI. BKM120 induced changes are derived using the formula (mean protein level post BKM120 minus mean protein level post vehicle)/mean protein level post vehicle treatment. Bar graphs are ordered based on %TGI from less to more sensitive to BKM120.

To assess whether there are short-term (3 days of therapy) treatment-induced proteomic changes that predict BKM120 response, we performed modest t test on each marker comparing samples treated with BKM120 versus vehicle. The number of significantly changed protein markers after BKM120 administration (adjusted *p* ≤ 0.05) ranged from as low as 18 in WHIM12, which is the most resistant PDX model, to as high as 105 in WHIM4, which is the most sensitive PDX model. The number of significantly changed protein markers was significantly positively correlated with %TGI (Figure 4). In addition, changes in several markers were associated with degrees of %TGI BKM120 (Figure 5). BKM120-induced reduction in the levels of CHAF1A and Caspase 3, pATR, DRIP130, EZH2, and Ki67 was associated with more effective tumor growth inhibition, while increased phosphorylation of Aurora kinases was associated with resistance (Figure 5).

## 3. Discussion

Triple negative breast cancer is a significant clinical challenge due to the lack of targeted therapy. Increasing evidence indicates the PI3K pathway as a potential therapeutic target in this disease. However, single agent PI3K inhibitors have shown modest anti-tumor activity [18]. To investigate biomarkers of response and molecular mechanisms of resistance, we tested 17 TNBC PDX models with varying genetic backgrounds and PI3K pathway signaling (Figure 1) for their tumor growth response to the pan-PI3K inhibitor BKM120. RPPA proteomic analysis of 182 markers at baseline and following short-term treatment (3 days) with either vehicle or BKM120 were analyzed to identify potential resistance biomarkers.

We demonstrated that PI3K inhibition induces varying degrees of tumor growth inhibition, with five models demonstrating over 80% TGI (Figure 2). BKM120 consistently reduced PI3K pathway activity, as demonstrated by reduced pAKT following therapy (Figure 3). Several protein markers showed significant association with resistance, including baseline levels of growth factor receptors (EGFR, pHER3 Y1197), PI3Kp85 regulatory subunit, anti-apoptotic protein BclXL, EMT (Vimentin, MMP9, IntegrinaV), NFKB pathway (IkappaB, RANKL), and intracellular signaling molecules including Caveolin, CBP, and KLF4 (Table 2, Figure S2), as well as treatment-induced increases in the levels of phosphorylated forms of Aurora kinases (Figure 5). Sensitivity was associated with higher baseline levels of proapoptotic markers (Bak and Caspase 3) (Table 2, Figure S2) and more markers being changed following BKM120 therapy (Figure 4). Interestingly, markers indicating PI3K pathway signaling activation at baseline were not significantly correlated to %TGI (Table S5). These results provide important insights in biomarker development for PI3K inhibitors in TNBC.

A strength of the study is the inclusion of a heterogeneous group of TNBC PDX models. For unbiased discovery, the study included unselected 17 TNBC PDX models, which were accrued based on availability. These models were similar to their human cancer counterpart (Figure S1) but were highly diverse in their genomic and proteomic profiles, TNBCtype, and with a wide range of PI3K pathway activities, as indicated by the RPPA PI3K activity score and levels of phosphorylated AKT, S6K, and PTEN expressions (Figure 1, Table 1). We noted that BL subtypes were fairly stable between human and different passages of PDX models; however, MSL and IM subtypes frequently converted to other subtypes (Table S2). Similar conversions have been observed in other studies [23], as a result of losing immune cells when passaged in immune-compromised mice [23]. The heterogeneity of this panel of TNBC PDX models is also reflected by the difference in %TGI to BKM120 (Figure 2) and the different proteomic responses (Figure 4) observed, although target inhibition has been uniformly observed across different PDX models (Figure 3).

Resistance mechanisms to PI3K inhibition in TNBC are complex due to numerous feedback loops and extensive crosstalk with other signaling pathways [24,25]. However, extensive proteomic analysis of clinical samples from patients enrolled in clinical trials of PI3K inhibitors is challenged by the ability to obtain sufficient tumor materials and the difficulty of obtaining serial samples, limiting biomarker development and the understanding of resistance mechanisms in individual patients. PDX models offer a unique resource for biomarker discovery. Several baseline biomarkers that were found to correlate with less responsiveness to BKM120 were reported previously in association with PI3K inhibitor resistance, for example upregulated RTK [26,27], EMT [28,29], and anti-apoptotic signaling [30]. However, other markers including PI3Kp85 regulatory subunit, NFKB pathway (IkappaB, RANKL), and intracellular signaling molecules including Caveolin, CBP, and KLF4 are also identified, which could represent potential novel resistance mechanisms. Further studies for validation are needed.

An interesting finding from this study is the significant association of treatment-induced upregulation of phosphorylated Aurora kinases in the less sensitive models. A recent study of kinome profiling of breast cancer cell lines using unbiased multiplexed inhibitor beads (MIBs), mass spectrometry (MS) demonstrated that resistance to drugs that target PI3K components, including AKT and mTOR, was associated with a failure to inhibit AURKA. The combination of the Aurora kinase inhibitor MLN8273 and PI3K-pathway inhibitor (GDC-0941, MK2206, or everolimus) was synergistic [31]. It was proposed that AURKA is upregulated by MYC and phosphorylates AKT, therefore leading to resistance to PI3K pathway inhibitors [31].

The limitation of the study is the small sample size of 17 PDX models. In addition, we have focused on RPPA of 182 markers important for oncogenic signaling. Furthermore, BKM120 is no longer in clinical development due to toxicity, and findings from this study will need to be validated with more clinically relevant PI3K inhibitors. However, despite these shortcomings, the results provided important biomarker candidates relevant to PI3K inhibitors in general. The study demonstrates the utility of the PDX model in investigating drug resistance mechanisms. More in-depth proteomic analysis and the testing of other clinical PI3K inhibitors are ongoing.

## 4. Materials and Methods

### 4.1. Chemicals and Antibodies

BKM120 (Cat. No. CT-BKM120) was purchased from Chemietek. The primary antibody for PTEN IHC was purchased from Cell Signaling Technology (Cat. No. 9559).

### 4.2. Generation of PDX Models

Fresh tumor specimens were obtained via biopsy or tumor resection from breast cancer patients after informed consent, in compliance with NIH regulation, institutional guidelines, and Institutional Review Board approval at Washington University [6]. Procedures for sample processing and establishment of orthotopic xenograft models in the 4th mammary fat mammary fat pad of NOD/SCID mice have been described in detail previously [6]. PDX models are available through the application to the Human and Mouse Linked Evaluation of Tumors Core [32]. 

### 4.3. Immunohistochemistry

Immunohistochemistry for PTEN was performed on 5-micron sections of formalin fixed paraffin embedded PDX tumors as described in our previous publication [33].

### 4.4. mRNA Gene Expression Analysis Using Agilent 4X44K Arrays

RNA was extracted from cryopulverized PDX tumor tissue according to established protocol and using [22]. Purified total RNA samples were then profiled using 4X44K human oligo microarrays (Agilent Technologies, Santa Clara, CA, USA) as previously described [34]. Raw Agilent 4x44K gene expression data were normalized using the BioConductor “limma” package sequentially through background subtraction, loess within-array normalization, and quantile between-array normalization. The probe level gene expression data were collapsed to gene level by median expression. ComBat was applied using the R package “sva” to the normalized gene expression data to correct for potential human/mouse batch effect [35]. Research use only PAM50 subtype classification were previously described [34]. Microarray data are available through the Gene Expression Omnibus (GEO) database (reference number GSE148949). TNBCtype was generated according to Lehmann et al. [36]. TNBC subtypes were assigned for each of the samples using “TNBCtype” [37,38,39].

### 4.5. Whole Exome Sequencing Analysis

The library was hybridized with the xGen Lockdown Exome Panel v1 (IDT Technologies) and sequenced using NovaSeq 6000 sequencing system. Adapter and low-quality sequences were trimmed from raw 2 × 150 bp paired end reads. The initially filtered reads were mapped to the GRCh38 human reference genome, and the validated bams (average sequencing coverage >40X) were used for downstream analysis and variant calling. For the PDX samples, the mouse reads were filtered using Disambiguate (v1.0) [40] before downstream analysis.

Somatic mutations were determined using our in-house SomaticWrapper pipeline [41], which uses several somatic variant calling tools including Strelka (v2.9.2) [42], Mutect (v1.1.7) [43], VarScan (v2.3.8) [44], and Pindel (v0.2.5) [45]. To generate high confident mutation callings, we used the mutations that were supported by at least 2 callers, cutoffs of at least 14 reads in the tumor and at least 8 in the normal. We used at least 4 supporting reads and variant allele frequency (VAF) 0.05 for mutations with a maximal VAF 0.01 in normal. In cases where normal tissue counterpart was not available, somatic variants were called using Mutect2 (v4.1.2.0) best-practice pipeline [46]. To reduce false-positive, we only used mutation sites having ≥ 20X coverage and >3 reads supporting mutations with ≥0.05 tumor VAF.

Somatic copy number alterations (SCNAs) in segment-level and gene-level were estimated using whole exome sequencing data; CNVkit v0.9.6 was performed on tumor-normal sequencing data [47]. Copy number of amplification or deletion regions were estimated via log2 ratio values (>0.9 or <−1.3, respectively).

### 4.6. Reverse Phase Protein Array (RPPA) and PI3K Signature Score

RPPA of PDX tumor lysates was performed at the Antibody-Based Proteomics Core at Baylor College of Medicine with methods described in previous publications [22]. Please see Table S3 for a complete list of the protein markers. PI3K signature score (PI3K_Score) is the sum of the phosphoprotein levels of Akt, mTOR, GSK3, S6K, and S6, minus the total levels of pathway-inhibitor PTEN [48].

### 4.7. Treatments of Patient-Derived Triple Negative Breast Cancer Xenograft Models

Seventeen TNBC PDX models with varying degrees of baseline PI3K pathway activities were selected from the Washington University Human in Mouse (WHIM) PDX collection [6] (WHIMs 2, 3, 4, 5, 6 10, 12, 13, 14, 21, 25, 29, 30, 31, 34, 36, 48). Early passages from each PDX model were propagated into the 4th mammary fat pad of 6-week-old NU/J homozygous female mice (Jackson lab, Cat. No. 2019). Treatment was initiated when the xenograft tumor reached approximately 5–7 mm in diameter. For the assessment of in vivo tumor growth response, tumor-bearing mice (6–8 mice per treatment group, 2 tumors per mouse, one each at the left or right 4th mammary fat pad) were treated with either vehicle or BKM120 (30 mg/kg) by oral gavage on days 1–5 of each week for 24–80 days. Two dimensional measurements (length and width) using Traceable Digital Calipers were performed 2–3 times each week. The following formula was used to calculate tumor volume: tumor volume (cm^3^) = (length × width^2^) × 0.5. Percentage tumor growth inhibition (%TGI) was calculated based on (1−[(Vt/V0)/(Ct/C0)])/(1−[C0/Ct]) × 100 [49]. Ct and C0 indicates tumor volume with vehicle control at time t (completion of 24–80 days of therapy) and time 0 (baseline), respectively. Vt and V0 represent tumor volume with BKM120 at time t (completion) and time 0, respectively. For treatment-induced proteomic changes in cell signaling pathways, mice were sacrificed on day 3, 2 h after administration of vehicle or BKM120 (*n* = 3 each treatment arm), and xenograft tumors were harvested and either snap frozen or fixed in 10% formalin. Snap frozen xenograft tumors were then pulverized to obtain tissue for nucleic acid preparation and RPPA analysis, as previously described [22].

### 4.8. In Vitro Cytotoxic Assay

Two TNBC cell lines, WHIM3 and MDA-MB-231, were tested for their cytotoxic response to BKM120 and an EGFR inhibitor (erlotinib or neratinib) either alone or in combination. WHIM3 cells were seeded at a density of 2000 cells per well in 96-well plates in RPMI-1640 medium with 10% fetal bovine serum (FBS) for 24 h prior to treatment. The cells were then treated with BKM120 (0, 0.25–2 uM) and erlotinib (0, 1.75–14 uM) or neratinib (0, 0.0625–0.5 uM), either alone or in combination. MDA-MB-231 cells were seeded at a density of 1500 cells per well in 96-well plates in DMEM/F-12 medium with 10% FBS for 24 h prior to treatment. The cells were then treated with BKM120 and neratinib either alone or in combination, with a concentration range of 0, 0.5–4 uM for both agents. Following 6 days of treatment, cell survival was assessed using Alamar Blue cell viability reagent. Each experiment was repeated thrice in triplicate for both cell lines. Synergistic or additive activity between the drugs tested was determined by calculating combination index values using the CalcuSyn software (Calcusyn software; Biosoft, Ferguson, MO, USA).

### 4.9. Statistical Analysis

The RPPA data were subjected to batch effect adjustment using the ComBat method [35] implemented in R package sva. Spearman correlation was calculated between a baseline protein marker and %TGI. LIMMA was applied to the RPPA data of each PDX model to identify the proteins differentiating BKM120-treated samples versus vehicle-treated samples [50]. The percent change in a protein was calculated as the difference between the estimated group means of BKM120-treated samples and vehicle-treated samples, divided by the absolute value of the group mean of the vehicle-treated samples. Significant differentially expressed proteins from LIMMA analyses were claimed at a false discovery rate adjusted *p* value <5%. Spearman correlation coefficient was calculated between the calculated percent change in a protein with %TGI and between the total number of LIMMA significant differential proteins and %TGI.

## 5. Conclusions

Using a panel of TNBC PDX models that represent a diverse genomic background and varying degrees of PI3K pathway signaling activities, we demonstrated the significant heterogeneity in the anti-tumor response of TNBC to PI3K inhibition. By analyzing the baseline and post-treatment PDX tumors, we were able to identify several potential resistance biomarkers as candidates for future preclinical and clinical validation. Importantly, our data also indicate that a loss of PTEN expression or elevation of AKT phosphorylation could not predict tumor responsiveness to PI3K inhibitor in TNBC. More in-depth proteomic analysis and the testing of other clinical PI3K inhibitors are ongoing.

## Figures and Tables

**Table 1:** Potential actionable mutations and genomic alterations in PI3K pathway genes.

PDX ID	Selected Actionable Mutations and Significantly Mutated Genes	PI3K Pathway Alterations
WHIM2, 5	PTCH2 p.W293S; JAK2 p.I166T; MAP3K8 p.P461L, TP53 p.Q167Tfs	AKT3 Amp
WHIM3	KRAS p.G13D; NF1 p.Q1775L	
WHIM4	TP53 p.E326Q, p.E285*, p.R175H; ALK p.R395H	PIK3CA amp
WHIM6		
WHIM10		
WHIM12	TP53 p.R248Q; PTPRJ p.K1017N; HRAS p.G12D; PIK3CA p.E109fs; PTEN p.T319*	PIK3CA p.V105_E109delinsT; PTEN p.T319*
WHIM13	TP53 p.C238Y; KIT p.D419H	
WHIM14	TP53 p.I195T, CSF1R p.N241K; NTRK3 p.T332M	
WHIM21	FGFR3 p.E135K, PTPRS p.P1506T; TP53 p.P151H	AKT1 Amp
WHIM25	TP53 p.R273H	
WHIM29	PTEN p.E284*, TP53 p.V216M	PTEN p.E284*
WHIM30	BRCA1 p.E1410* (germline); TP53 p.X125_splice site; ATM p.D126E; ATR p.M211T	
WHIM31	BRCA1 p.3604delA (germline); TP53 p.R342*, KRAS p.A134V; PIK3CG p.I287M	
WHIM34	TP53 p.R248W; PTEN p.D310G, GATA3 p.T421Rfs*55	PTEN p.D310G
WHIM36	TP53 p.F134C;	
WHIM48	TP53 p.Y205H; MET p.R988C	

**Table 2:** Baseline protein markers of response to BKM120.

Protein Marker	SpearmanCorr2TGI	*p* Value
MMP9	−0.70098	0.002347
EGFR	−0.64461	0.006396
Integrina5	−0.57843	0.016803
Caspase 3	0.541667	0.026735
p-IkappaB (S32/36)	−0.5098	0.038642
RANKL	−0.48284	0.051624
p-HER3(Y1197)	−0.47304	0.057094
Caveolin	−0.46569	0.061477
CBP	−0.46324	0.062994
PI3Kp85	−0.45588	0.067714
Bak	0.443627	0.076168
Bcl.xL	−0.43873	0.079763
Vimentin	−0.43137	0.085389
KLF4	−0.42892	0.087329

## Supplementary Materials

The following are available online at https://www.mdpi.com/2072-6694/12/12/3857/s1, Figure S1: Correlation of somatic mutations between PDX samples and human tumor samples; Figure S2: Correlation of baseline tumor RPPA protein markers with %TGI; Figure S3: Spearman’s correlation of baseline protein levels with %TGI is indicated for each marker. Table S1: Clinical Characteristics of Patients Corresponding to TNBC PDX Models, Table S2: TNBC Subtypes of PDX models, Table S3: RPPA list of protein markers See attached excel spreadsheet, Table S4: Significantly changed markers following treatment with BKM120 compared to vehicle, Table S5: Baseline PI3K protein markers by RPPA in correlation to %TGI.
