# Supplementary material for: Proteomic Resistance Biomarkers for PI3K Inhibitor in Triple Negative Breast Cancer Patient-Derived Xenograft Models
Source: Cancers (Basel). 2020 Dec 21;12(12):3857. doi: 10.3390/cancers12123857 (PMC7765949; doi:10.3390/cancers12123857)
Supplement: Supplementary file 1 [file cancers-12-03857-s001.zip › cancers-1026672-supp-final/cancers-1026672-supp-final.pdf]

Supplementary Materials: Proteomic Resistance Biomarkers for PI3K Inhibitor in Triple Negative Breast Cancer Patient-Derived Xenograft Models

Zhanfang Guo, Tina Primeau, Jingqin Luo, Cynthia Zhang, Hua Sun, Jeremy Hoog, Feng Gao, Shixia Huang, Dean P. Edwards, Sherri R. Davies, Rebecca Aft, Li Ding, Matthew J. Ellis, Shunqiang Li and Cynthia X. Ma

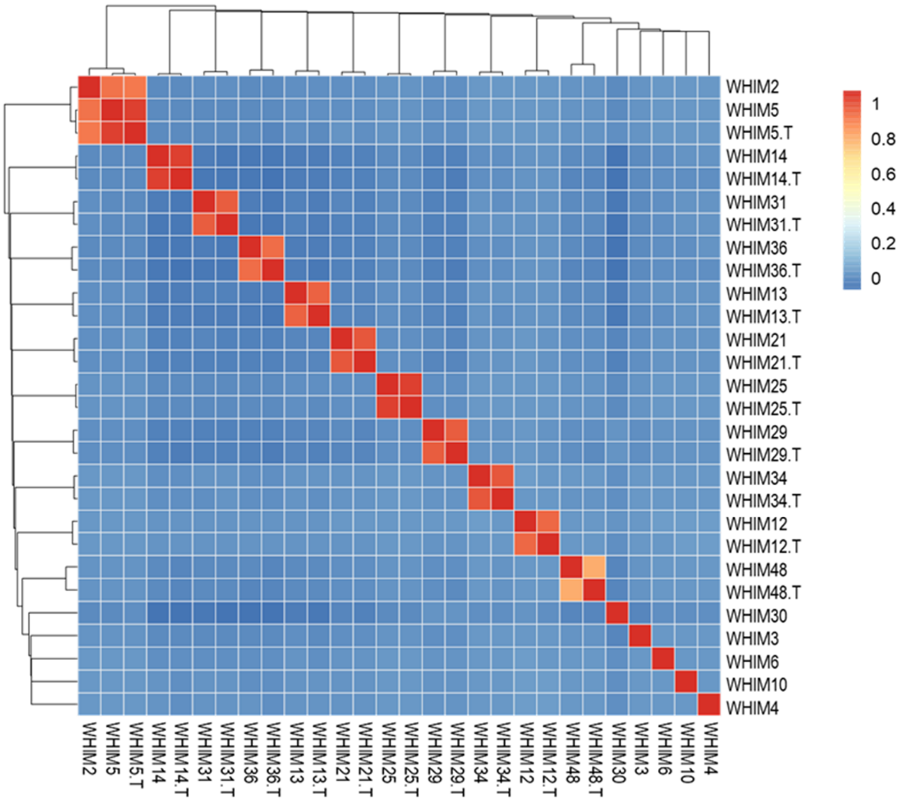

Figure S1. Correlation of somatic mutations between PDX samples and human tumor samples. The color scale on the right means pearson correlation coefficient. The sample name with "T" means human tumor sample.

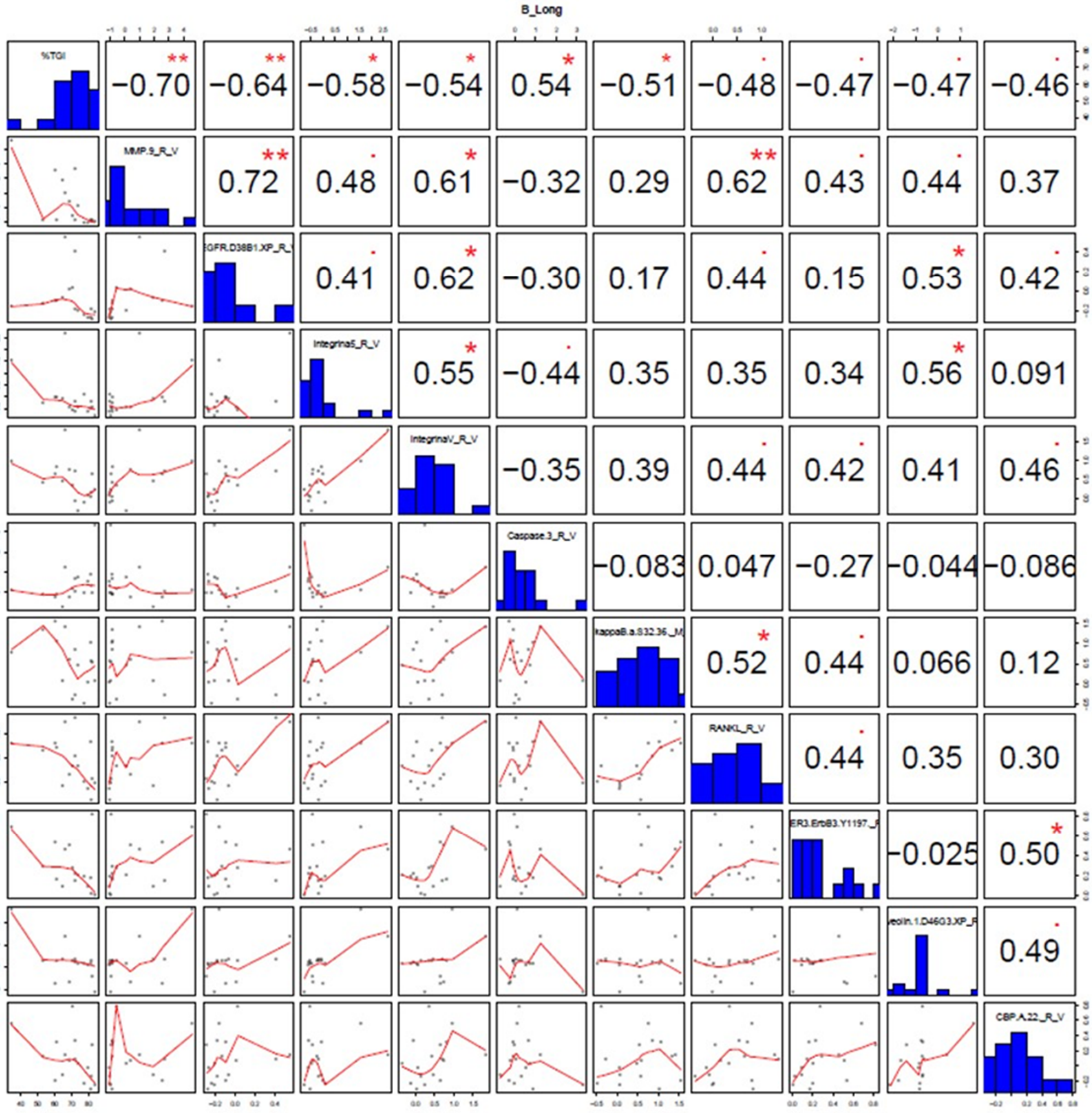

Figure S2. Correlation of baseline tumor RPPA protein markers with %TGI. Spearman Correlation of baseline protein levels with %TGI is indicated for each marker.

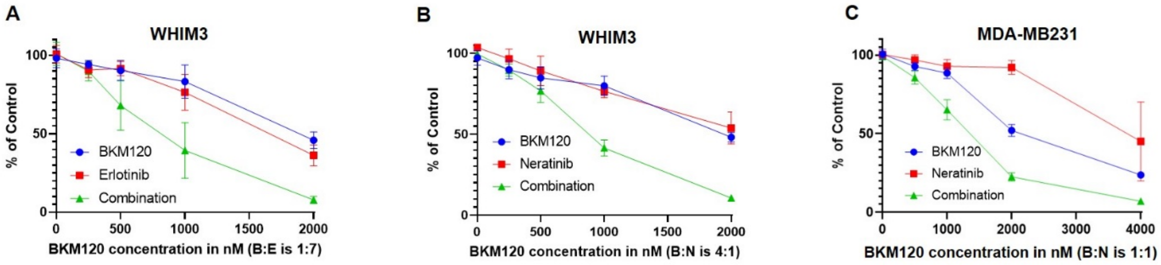

	ED50	ED75	ED90
CI	0.70	0.52	0.39

	ED50	ED75	ED90
CI	0.66	0.45	0.30

	ED50	ED75	ED90
CI	0.76	0.69	0.62

Figure S3. Synergistic cytotoxic effect of BKM120 in combination with an EGFR inhibitor in TNBC cell lines in vitro. % cell survival compared to control as measured by Alamar Blue following 6 days of treatment with increasing concentrations of BKM120 and erlotinib in WHIM3 (Panel **A**), BKM120 and neratinib in WHIM3 (Panel **B**) or MDA-MB 231 (Panel **C**), either alone or in combination, are shown. X axis indicates the concentrations of BKM120. The indicated EGFR inhibitor is tested at fixed drug concentration ratio in relation to BKM120. The combination index was shown for each combination at ED50, ED75 and ED90. BKM120; E, erlotinib; N, neratinib.

Table S1. Clinical Characteristics of Patients Corresponding to TNBC PDX Models.

PDX ID	Age (Race)	Tissue source (*stage providing samples)	*Path stage at diagnosis	DFS (mon.)	OS (mon.)	Patient treatment history	
						Pre Engraftment	Post Engraftment
**WHIM2**	**44 (AA)**	**Breast (cIIIA)**	**IIIA**	**5.7**	11	None	AC-T (neo)
WHIM3	62 (CA)	Breast (IV)	IV	NA	3.4	None	Gem
WHIM4	49 (AA)	Skin (IV)	pCR	4	37	AC-TH (neo), Dox/Gem/H, Cap, D/Bev	D/Bev
WHIM5	44 (AA)	Brain (IV)	IIIA	5.7	11	AC, T	None
WHIM6	50 (AA)	Breast (IV)	IV	NA	36	None	FEC, T, Cap, Dox, Gem, Nav
WHIM10	52 (CA)	Skin (IV)	IA	11	48	FEC (adj), T (adj)	Bev/D, Bev/T
WHIM12	65 (CA)	Breast (IIB)	IIB	9.6	12	None	FEC, T, Bev, Carb
WHIM13	42 (CA)	Skin (IV)	IIA	8	45	AC-D (neo), T/Bev	Cap, Dox, T/Bev
WHIM14	41 (CA)	Skin (IV)	IIB	25	38.9	FEC-T (neo)	None
WHIM21	64 (CA)	Breast (IIB)	IIB	0.9	8	AC-T (neo)	Cap, Ixa
WHIM25	49 (AA)	Skin (IV)	IIIC	1	16	T/Bev-AC/Bev (neo)	UCN-01/Irinotecan
WHIM29	46 (CA)	Breast (IV)	IV	NA	2	None	None
WHIM30	35 (CA)	Breast (cIII)	pCR	>75	>82	None	pCR to AC, T
WHIM31	48 (CA)	Skin (IV)	IIIA	80	115	AC-D (adj), Carb/T, Ana, Tam, Ful, Cap/Bev, Nav/Gem, Carb/Gem, CMF, Eri	A
WHIM34	53 (CA)	Skin (IV)	IIA	56	65	None	T
WHIM36	43 (CA)	Skin (IV)	IIIC	13	42	AC (neo), T (adj), Iniparib/Carb/Gem	Cap, C
WHIM48	49 (CA)	LN (IV)	III	18	37.8	TAC (adj), Cap, T/Bev, Eri, CM	None

DFS, time from breast surgery to recurrence; OS, time from diagnosis to death; c, clinical; *staging is per AJCC version 7; AA, African American; CA, Caucasion American; LN, lymph node; neo, Neoadjuvant; adj, Adjuvant; NA, not applicable; A, Adriamycin; C, Cyclophosphamide, T, Paclitaxel; Gem, Gemcitabine; Cap, Capecitabine; Dox, Doxil; D, Docetaxel; Bev, Bevacizumab; Nav, Navelbine; E, Epirubicine; F, 5-FU; Carb, Carboplatin; Ixa, ixabepilone; H, Herceptin; Ana, Anastrozole; Ful, Fulvestrant; Eri, Eribulin; Tam, Tamoxifen; M, methotrexate.

Table S2. TNBC Subtypes of PDX models.

PDX ID	Human Tumor	PDX Passage				
		1	2	3	4	5
WHIM2	MSL	LAR		LAR		
WHIM3			BL2			
WHIM4	BL1	BL1		BL1	BL1	BL1
WHIM5	M	UNS		UNS		UNS
WHIM6	IM	IM		BL2		BL2
WHIM10		BL1		BL1	BL1	
WHIM12	IM	MSL		UNS		
WHIM13	M	M	M			
WHIM14	M		M			
WHIM21	MSL	BL1	BL1			
WHIM25	MSL	BL1				
WHIM29	UNS	LAR	LAR			
WHIM30		IM				
WHIM31	BL2	BL1	BL1			
WHIM34	M					M
WHIM36	BL1		BL1			
WHIM48	IM	M				

UNS: unspecified; WHIM3, 10 and 30 had insufficient tumor RNA for microarray analysis.

Table S3. RPPA list of protein markers See attached excel spreadsheet.

Table S4. Significantly changed markers following treatment with BKM120 compared to vehicle.

Biomarkers	Average_ logFC	Average_ %Change	Average_ adj.P.Val.Ttest	Average_ Rank	Significant WHIM models	FisherCombined TestP	FisherCombined TestP_FDRadjusted
**p-Akt(S473)**	-0.61	-1.14	0.04	22.5	2;3;4;5;6;12;14;20;21;25;29;30;31;34;48	8.39E-132	1.53E-129
**p-p70S6K(S371)**	-0.59	-3.11	0.07	37.7	2;3;6;10;13;14;20;21;25;29;30;31;34;48	9.70E-40	2.21E-38
**p-Akt(T308)**	-0.49	-1.25	0.09	37.8	2;3;5;6;12;14;20;21;25;29;30;31;34;36	8.93E-76	8.12E-74

Average_logFC: LIMMA log2 fold change averaged across 17 PDX models. Average % change: % change=(group mean of BKM120-group mean of vehicle)/absolute (group mean of vehicle), averaged across 17 PDX models .Average_adj.P.Val.Ttest: LIMMA FDR adjusted P value comparing BKM120 to Vehicle, averaged across 17 WHIM lines. Significant WHIM models: the WHIM line where the protein was significant in LIMMA analysis. FisherCombinedTestP and FisherCombinedTestP_FDRadjusted P: the Fisher's combined P value was calculated across WHIM lines using the raw and FDR-adjusted P values, respectively.

Table S5. Baseline PI3K protein markers by RPPA in correlation to %TGI.

Protein Markers	SpearmanCorr2TGI	*P* value
Akt	−0.375	0.138717
p-Akt(S473)	−0.30147	0.238965
p-Akt(T308)	−0.33824	0.184211
p70S6K	−0.18382	0.478639
p-p70S6K(T412)	−0.1348	0.605275
p-p70S6K(S371)	−0.05882	0.824102
p-mTOR(S2448)	−0.28186	0.272097
mTOR	−0.29412	0.251066
PTEN	0.139706	0.59201

© 2020 by the authors. Submitted for possible open access publication under the terms and conditions of the Creative Commons Attribution (CC BY) license (http://creativecommons.org/licenses/by/4.0/).
